# Predictors of Late HIV Diagnosis among Adult People Living with HIV/AIDS Who Undertake an Initial CD_4_ T Cell Evaluation, Northern Ethiopia: A Case-Control Study

**DOI:** 10.1371/journal.pone.0140004

**Published:** 2015-10-08

**Authors:** Melkamu Bedimo Beyene, Habtamu Bedimo Beyene

**Affiliations:** 1 Department of Public Health, College of Health Sciences, Bahir Dar University, Bahir Dar, Ethiopia; 2 Department of Microbiology, Immunology and Parasitology, College of Health Sciences, Addis Ababa University, Addis Ababa, Ethiopia; University of Athens, Medical School, GREECE

## Abstract

**Introduction:**

Early HIV testing and timely initiation of ART is critical for the improved quality of life of PLWHIV. Having identified a higher rates of Late HIV diagnosis, this study was aimed to determine Determinants of late diagnosis of HIV among adult HIV patients in Bahir Dar, Northern Ethiopia.

**Methods:**

A case control study was conducted between January 2010 to December 2011 at Bahir Dar Felege Hiwot Referral Hospital. The study subjects consisted of 267 cases and 267 controls. Cases were adult people living with HIV/AIDS whose initial CD_4_ T cell count was < 200/μl of blood. Controls were those with a CD_4_ T cell count of greater than 200/ μl. Trained staff nurses were involved in data collection using a semi-structured questionnaire. Data was entered and analyzed using SPSS version 20. Descriptive statistics and Binary logistic regression were performed.

**Results:**

Subjects who hold a certificate and above (AOR = 0.26; 95% CI = 0.13. 0.54), being initiated by friends, families and other socials to undertake HIV testing (AOR = 0.65; 95% CI = 0.29, 1.48), who reported a medium and high knowledge score about HIV/AIDS and who undertake HIV testing while visiting a clinic for ANC (AOR = 0.40; 95% CI = 0.19, 0.83) were less likely to be diagnosed late. Subjects who undertake HIV testing due to providers’ initiation (AOR = 1.70; 95%CI = 1.08, 2.68), who reported a medium internalized stigma (AOR = 4.94; 95% CI = 3.13, 7.80) and who reported a high internalized stigma score towards HIV/AIDS (AOR = 16.64; 95% CI = 8.29, 33.4) had a high odds of being diagnosed late compared to their counterparts.

**Conclusion:**

Internalized stigma, low knowledge level about HIV/AIDS, not to have attended formal education and failure to undertake HIV testing by own initiation were significant determinant factors associated with Late HIV diagnosis. Education about HIV/AIDS, promotion of general education, and encouraging people to motivate their social mates to undertake HIV testing are highly recommended.

## Introduction

With more than 1.5 million people living with HIV/AIDS (PLHIV), Ethiopia is one of the countries seriously affected by the epidemic [1]. In the last few years, the Government of Ethiopia has increased efforts to accelerate progress towards universal access to HIV prevention, treatment and care [2]. Despite such an increased access to HIV/AIDS testing, counseling, prevention, care and treatment services; studies show that people are still diagnosed late and as such initiated on ART late losing advantages of early initiation of ART.

During the natural course of HIV infection, there is a progressive loss of CD4 T cells to an average of 60–100 cells/uL per year [3–5]. A reduction in CD4 T cells below 200 cells/uL makes the host highly susceptible to opportunistic infections and increases overall AIDS related morbidity and mortality. It is universally documented that, in the absence of effective antiretroviral therapy, most people infected with HIV will progress to AIDS in an approximately ten years. This period varies from patient to patient based on host and viral factors [6]. Survival in human immunodeficiency virus (HIV) infected individuals have improved with the introduction of highly active antiretroviral therapy (HAART) [7–9].

Testing, diagnosis, and medical care soon after HIV infection or before patients develope opportunistic infections and other AIDS defining illness(ADIs) and clinical AIDS, can prevent illness, improves survival, and reduce transmission at large. However, patients receiving HIV diagnosis late in the course of infection at a more severely immune-compromised stage are more likely to present with co-morbidities like tuberculosis, and have short-term mortality[10].

Delay in diagnosis is significant to both disease prognosis at patient level and transmission at community and public health level. An early diagnosis provides opportunities for reducing or halting further transmission due to changes in risk behavior. While early diagnosis of HIV has got such a benefit, many current findings across the world show that people are still diagnosed late for HIV. A study at felege hiwot referral hospital ART clinic in Bahirdar, northwest Ethiopia; (our current study area) showed a 60% prevalence of late HIV diagnosis [11, 12]. The predictors of late HIV diagnosis are also somehow different among literatures, from different areas and they are not sufficiently studied in our study area. So, this quantitative case control study was designed to determine factors associated with late HIV diagnosis.

## Methods and Materials

### Study area and design

An institution based quantitative case control study was done from May 2012 to April, 2013 at Bahirdar Felegehowot Referal Hospital (FHRH); the only referral hospital of the town, Northern Ethiopia. We preferred the case control design because it is thought that the case control study design is more powerful to study factors or predictors. Bahirdar is the capital city of Amhara National Regional State (ANRS) and it is located 562kms from Addis Ababa. This general service public institution provides this service (CD4 counting) as one part of the HIV/AIDS care and treatment services to monitor disease progression and hence check eligibility for initiation of ART. Currently as of the report from the ART clinic section head of this hospital; the referral hospital evaluates on average about seven cases presenting for initial CD4 count per day.

The study population for this study included those adult ARV clients who visited FHRH for initial CD4 count during two years; January 1, 2010 to December 31, 2011. These recent two years population is intentionally preferred to minimize recall bias on one hand and to see the current situation by considering the recent population on the other hand.

### Sample size determination

The sample size for this study was determined by using a formula for unmatched case control study for detecting the different effect sizes of the predictor variables independently. EPI-INFO version.7 statistical software was used by assuming cases to control ratio of 1:1, a power of test 80% and confidence level of 95% for studies with full information for sample size calculation

The maximum sample size was obtained considering a study in Spain. The predictor variable that yielded the maximum sample size was educational level measured as low educational level and high educational level. So on this study prevalence of low educational level among the cases was among 42% that in the controls was about 30% and hence the AOR was 1.69 yielding the sample size of 534 [13]. So 267 cases (people with delayed HIV diagnosis) and 267 controls (people who were not delayed) have been considered

### Sampling method and procedures

Bahir Dar Felege Hiwot Referal Hospital was selected as it was the only public referral hospital of the town. For this case control study a risk set sampling technique was used. This is one of the sampling techniques used in case control study design where for every case found the next control was considered and the sampling procedure goes that way until the sample size was full.

The sample selection started on August 01; 2012 and extended until the number of cases and controls thought were fulfilled. We started selecting sample and collecting data on august 01; 2012 because we planned to assess late diagnosis using the CD4 count obtained within six months of diagnosis and those who could have possibly been diagnosed on December 31, 2011 shall be awaited until end of July 2012.

Identification of cases and controls was done by the principal investigator with the help of the ARV clinic nurses. Those who had been diagnosed positive for HIV earlier than six months from the date they come for initial CD4 count evaluation have not been included in the study. This was because the baseline CD4 count (CD4 count at diagnosis) was operationalized for this study to be the first CD4 count within six months of diagnosis of HIV. Those who are lost to follow up have also been excluded from the study since it is difficult to terrace them in our setting.

### Data collection

A semi-structured questionnaire was prepared. Three ART nurses were recruited to handle the data collection task. Data collectors had been given a two days training on the sampling procedure and to make the question items understood uniformly by the data collectors. The questionnaire was pre-tested on 5% of study subjects. The principal investigator supervised every aspect of the data collection as a head supervisor and the ART nurse mentor at the hospital had been shouldering the regular supervision task. The filled questionnaires were always gathered to/by the principal investigator and/or the supervisor on daily basis.

The data were collected on Patients Socio-demographic and economic variables(Age, Sex, Educational status, Religion, Marital status, Occupation, Place of residence, Living with Family, Employment status, Monthly income of the family), Personal and behavioural factors(Fear of stigma and discrimination, Attitude towards the HIV, VCT and the treatment, Knowledge towards HIV/AIDS, care and treatment services available, place of HIV testing, as to when to undertake testing, how decided HIV testing, whom initiated HIV testing.

Moreover, clinical and laboratory information at or not later than 6 months of initial HIV testing such as CD4 cell counts and WHO clinical staging were recorded. The initial (first) CD4 count was the one that the patient has obtained within six months of diagnosis of HIV.

Knowledge about HIV/AIDS was measured using a validated tool consisted of a set of 18 questions to assess knowledge of the clients about HIV/AIDS and then; those who have responded correctly less than fifty percent of the questions are categorized as having low level of knowledge, those answering correctly 50% to 74.99% of the questions are categorized as having medium level knowledge about HIV /AIDS and those answering correctly for ≥75% of the questions are categorized as having high level of knowledge about HIV/AIDS [14]. Internalized stigma towards HIV/AIDS was measured using 22 Likert scaled question items developed for this purpose [15]. The scores for each respondent for all the 22 questions were added and then divided for 22 to get average attitude score. Then; those with average attitude score of less than 2.00 are categorized as having low internalized stigma score, those having average score from 2.00 to 3.00 as having medium internalized score and those with average score greater than 3.00 as having high internalized stigma score.

### Data processing and analysis

Data from corrected questionnaires were coded and then entered to statistical packages, specifically SPSS v.16. The data were also analysed using SPSS v.20. Basic descriptive analyses were done. Logistic regression was fit to find out determinants of late diagnosis of HIV. Bivariate logistic regression was first be fitted and those independent variables which become significant on the bivariate regression at 20% level of significance were included in the multiple logistic regression analysis. Backward Stepwise Multiple logistic regression was fitted to determine the net effect of each explanatory variable on late diagnosis of HIV; using 5% level of significance as a cut point for entry and 10% level of significance as a cut point for removal.

### Ethical considerations

This study was approved by Institutional Ethics Committee/IEC of college of medicine and health sciences, Bahirdar University. Ethical clearance letter was obtained from same institution. A written informed consent was obtained from study subjects who were able to read and write and for those who were illiterate study participants; the data collectors inform each respondent and confirmed the willingness by signing on the informed consent sheet. And the institutional Ethics Committee of Bahir Dar University approved this consent procedure. To maintain the confidentiality of the patients’ data, staff nurses working at the respective ART clinics were preferred to collect the data. Name and other personal identifiers had not been written on the questionnaire.

## Results

### Socio-demographic and economic characteristics of respondents

A total of 534 subjects (267 cases and 267 controls) were included in this study of whom 250(46.8%) were males and 284(53.2%) were females. Among the cases 44.6% were males and the rest 55.4% are females. Among the controls 49.1% were males(**Table 1).**

**Table 1:** Socio-demographic and economic characteristics of Adult PLHA who had undertaken initial CD4 count at Bahirdar FHRH, January 2010 to December 2011.

Variables	Categories	Cases		Controls	
		n	%	n	%
**Sex**	Male	119	44.6	131	49
	Female	148	55.4	136	51
**Age in years**	Less than 27	49	18	90	34
	27.00–31	78	29	63	24
	32.00–39.00	59	22	54	20
	≥40	75	28	56	21
**Marital status**	Never married	50	18.7	41	15.3
	Married	145	54.3	150	56.1
	Divorced	3	1.1	2	0.7
	Whose spouse has died	39	14.6	50	18.7
	Widowed	21	7.9	24	8.9
**Residence**	Bahirdar	214	80.1	209	78.3
	Out of Bahirdar	53	19.9	58	21.7
**Formal educational status**	No formal education	50	18.7	32	11.9
	Education up to 2^0^ level	158	59.2	145	54.3
	Certificate, diploma and above	59	22.1	90	33.7
**Occupation**	Farmer	18	6.7	18	6.7
	Merchant	63	23.6	79	29.5
	Government employee	97	36.3	71	26.5
	Housewife	35	13.1	49	18.3
	Private employee	29	10.8	20	7.5
	Daily laborer	25	9.3	30	11.2
**Monthly income**	<500 ETB	56	20.9	62	23.2
	500–999 ETB	70	26.2	77	28.8
	1000–1499 ETB	65	24.3	60	22.5
	1500–1999 ETB	44	16.4	35	13.1
	> = 2000 ETB	32	11.9	33	12.3

### Clients’ immunological, clinical and behavioral characteristics

The baseline CD4 count of the clients was generally right skewed with a range of 1308. About half (50.5%) of the subjects in this study presented with WHO stage III clinical stage at the time they came for initial CD4 count evaluation.

As shown in (**Table 2)** 228 (42.8%) of respondents decided to undertake HIV testing by their own initiation where as 213 (40.0%) being initiated by health professionals at the hospital when the respondents visited the hospital for other health services such as provider initiated testing and counseling (PITC).

**Table 2:** Immunological, clinical and behavioral characteristics of adult PLHA who undertake initial CD4 count at Bahirdar FHRH, Northern Ethiopia.

Variables		Cases		Controls	
		n	%	n	%
**WHO stage**	Stage I	25	9.3	54	20.2
	Stage II	58	21.7	91	34
	Stage III	159	59.6	109	40
	Stage IV	25	9.3	13	4.8
**Discussion with other people about HIV**	Yes	99	37	86	32.2
	No	168	63	181	67.8
**How decided testing for HIV**	Self-initiation	113	42.3	115	43
	Friends, families/socials	16	5.9	25	9.3
	PITCH	119	44.5	94	35.2
	While visiting for ANC	19	3.1	32	12
**Place of first HIV test**	Government health facility	195	73	215	80.5
	Private health facility	51	19.1	36	13.5
	NGOs’ health facilities	12	4.4	9	3.3
**Was the test for the first time**	Yes	235	88.7	246	92.1
	No	30	11.3	21	7.9
**When should one undertake HIV testing**	When feeling sick	35	13.1	44	16.5
	Before marriage	48	18	32	12
	Requested by professional	5	1.8	10	3.7
	As planned by oneself	169	63.3	159	59.6
	Don’t know about this	6	2.2	19	7.1
**Used to think that one should undertake testing seeming apparently healthy**	Yes	160	60	143	53.6
** **	No	107	40	124	46.4
**Heard about ART**	Yes	190	71.4	176	65.9
	No	76	28.6	91	34.1
**Knowledge about higher survival while taking ART**	Yes	153	57.8	140	52.6
** **	No	112	42.2	126	47.4
**Know about higher survival while taking ART early**	Yes	141	52.8	120	44.9
	NO	126	47.2	147	55.1
**Who should undertake HIV test**	Everybody by plan	82	30.7	58	21.7
	Not everybody; but those at higher risk	185	69.3	209	78.3
**Clients knowledge score about HIV/AIDS**	Low knowledge score	74	27.9	51	33.2
	Medium knowledge score	97	36.6	95	60.1
	High knowledge score	94	35.5	12	7.6

PITCH: Provider initiated testing and counseling for HIV

Majority of the respondents (62.2%) replied that they used to think that people should undertake HIV testing regularly at some intervals of time as planned by the individual himself. In this study, only 303 (56.7%) of respondents mentioned that people should undertake HIV testing while seeming apparently healthy. The remaining 43.3% reported that they had not used to think that it was necessary to undertake testing while apparently healthy. Moreover, 68.7% of the respondents had already heard about ART before they get tested and known their HIV status. A 293 (55.2%) already knew about higher survival advantage while taking ART before they had been counseled. Similarly 261 (48.9%) of the respondents already knew a better survival rate if ART is taken earlier (**Table 2**)

### Respondents’ knowledge about HIV/AIDS and their internalized stigma towards their HIV positive status

The knowledge of individuals about HIV/AIDS was measured using a tool to assess brief knowledge of clients about HIV/AIDS [14]. Using this measurement 125 (23.5%), 192 (36.2%) and 214 (40.3%) of the respondents had a low, medium and high knowledge score about HIV AIDS respectively. A low knowledge level was more prevalent among the cases than the controls and medium level of knowledge was almost equally prevalent between the two categories (97 by 95). A high level of knowledge was slightly more prevalent among the controls than the cases (94 by 120) indicating the likely association between knowledge about HIV/AIDS and delayed diagnosis of HIV.

Internalized stigma about their HIV status was measured using twenty two question items taken from Multidimensional Measure of Internalized HIV Stigma tool [15]. According to this measurement 192 (36.2%) of the clients had low internalized stigma and 254 (47.8%) had medium level stigma towards HIV/AIDS. Eighty five (16%) had high internalized stigma towards HIV/AIDS. The cross tabulation of stigma score with delayed diagnosis of HIV showed that most of the controls had low internalized stigma.

#### Factors associated with delayed HIV diagnosis among adult People undertaking initial CD4 count at Bahir Dar FHRH, North Ethiopia

From a multiple regression analysis it is shown that, educational status was significantly associated with a delay in HIV diagnosis. To be a holder of certificate, diploma or above had a low odds of being tested late (AOR = 0.26; 95% CI = 0.13, 0.54; P value < 0.001). Moreover, those who undertake HIV testing being initiated by friends, families and other socials were less likely to be diagnosed late than those who undertook the HIV testing by themselves. In contrary, subjects who undertake HIV testing due to health service providers’ initiation were more likely to be diagnosed late than those who undertook the test by their own initiation (AOR = 1.70; 1.08, 2.68; P value = 0.023).

Those who undertake the test while visiting the health service for antenatal care (ANC) were less likely to be diagnosed late. Knowledge about HIV/AIDS and internalized stigma towards HIV/AIDS had also been significantly associated with a delay in HIV diagnosis. Clients with a medium knowledge level were about 2 times more likely to be diagnosed early and those with a high knowledge score were about 3.5 times more likely to be diagnosed early compared to subjects with low knowledge level. On top of this, subjects with a medium internalized stigma were 5 times more likely to be diagnosed later and those reporting a high internalized stigma score were 16 times more likely to undertake HIV diagnosis late than their counter parts (**Table 3**). But age, occupational status, and place of first HIV test has not shown any significant association with late HIV diagnosis

**Table 3:** Factors associated with delayed diagnosis of HIV among Adult PLHA who had undertaken initial CD4 count at Bahirdar FHRH from January 2010 to December 2011.

Variables	Delayed HIV Diagnosis.		Crude odds ratio (95% CI)	Adjusted odds ratio (95% CI)	P-value
	Case	Control			
**Age**	261	263	1.02(1.001,1.04)		
**Educational status**					
No education	50	32	1.00	1.00	**<0.001**
Any to at most 2^0^ school	158	145	0.7 (0.42, 1.15)	0.63 (0.34, 1.18)	0.141
Certificate, Diploma and above	59	90	0.42 (0.24, 0.73)	0.26 (0.13. 0.54)	**<0.001**
**Occupation**					
Farmer	18	18	1.00		
Merchant	63	79	0.78 (0.38, 1.66)		
Government employee	97	71	1.37 (0.66, 2.81)		
House wife	35	49	0.71 (0.33, 1.57)		
Private employee	29	20	1.45 (0.61, 3.45)		
Daily labourer and others	25	30	0.83 ().34, 1.93)		
**How decide testing for HIV**					**0.001**
By self initiation	113	115	1.00	1.00	
Being initiated by friends, families and other socials	16	25	0.65 (0.33, 1.28)	**0.65 (0.29, 1.48)**	**0.303**
PITCH	119	94	1.29 (0.89, 1.87)	**1.70 (1.08, 2.68)**	**0.023**
While visiting for ANC	19	32	0.60 (0.32, 1.13)	**0.40 (0.19, 0.83)**	**0.014**
**Place of testing**					
Government health facility	195	215	1.00		
Private health facility	51	36	1.56 (0.98, 2.50)		
NGOs’ health facility	12	9	1.47 (0.61, 3.57)		
**Was it first test**					
Yes	235	246	1.00		
No	30	21	1.50 (0.83, 2.69)		
**Heard about ART**					
Yes	190	176	1.00		
No	76	91	0.77 (0.54, 1.12)		
**Know about higher survival while taking ART early**					
Yes	141	120	1.00		
No	126	147	0.73 (0.52, 1.03)		
**Knowledge about HIV/AIDS**					**<0.001**
Low level of knowledge	74	51	1.00	1.00	
Medium level of knowledge	97	95	0.70 (0.45, 1.11)	**0.46 (0.26, 0.81)**	**0.007**
High level of knowledge	94	120	0.54 (0.35, 0.84)	**0.28 (0.15, 0.50)**	**<0.001**
**Internalized stigma towards HIV/AIDS**					**<0.001**
Low internalized stigma score	47	145	1.00	1.00	
Medium internalized stigma score	151	103	4.52 (2.99, 6.84)	**4.94 (3.13, 7.80)**	**<0.001**
High internalized stigma score	69	16	13.31 (7.05, 25.12)	**16.64 (8.29, 33.40)**	**<0.001**

## Discussion

This case control study assessed factors associated with late diagnosis of HIV. The analysis for these factors in our study identified several important findings. The major predictor variables for late HIV diagnosis among the adult PLHIV were perceived medium to high internalized stigma, low awareness about HIV/AIDS and being at low educational level. Other factors such as not to have initiated by social mates and waiting PIHCT to undertake HIV testing were also found significantly associated with late HIV diagnosis.

Several definitions have been used to date for “late diagnosis”. Some define delayed diagnosis of HIV when the diagnosis of an AIDS defining condition (ADC) occurs either before or concomitantly to an HIV diagnosis [16–18], during the subsequent six months [19, 20], or during the following year of HIV diagnosis [21,22]. Other definitions used CD4 cell count: less than 200 cells/μl [23–25], or less than 350 cells/μl [26]. Finally, combinations of both low CD4 cell count and AIDS defining conditions [18, 20–22, 27] have been also considered late HIV diagnosis.

For this study delayed HIV diagnosis is defined as diagnosis of HIV at CD4 counts less than 200 cells/μl. The baseline/initial CD4 count (CD4 count at diagnosis of HIV) will be considered to define cases and controls (whether it is late diagnosis or not) when patients are having their initial CD4 cells counted within six months of diagnosis of HIV. Educational status, the motive that initiated the clients to undertake HIV testing, knowledge about HIV/AIDS and internalized or perceived stigma towards HIV/AIDS had been identified significantly associated factors with late HIV diagnosis.

In the present study, those who had attended education beyond secondary school were less likely to be diagnosed late than those who had not attended any formal education adjusting for the effect of other factors in the model. A significant difference in late diagnosis existed between those who have not attended any education and those who have attended beyond secondary education. This was consistent with finding such as in Spain, where an institution based study showed delayed diagnosis of HIV associated with low educational status [18]. This finding however, couldn’t be compared with the study in South Wollo in Ethiopia because the study in Wollo had not assessed the effect of education status on late presentation [28]. Our finding was also in line with reports from Venezuela and Indian HIV cohorts where a low educational level was shown to be among the main barriers to late diagnosis of HIV [29, 30].

In this study, motive that initiated the clients to undertake HIV testing was significantly associated with delayed diagnosis of HIV. Those who undertook HIV testing being initiated by friends, families and while visiting the health facility for ANC were less likely to be diagnosed late than those who undertake the test by their self initiation. But those who undertake the test by being initiated by health professionals or due to PITCH while the respondents come being sick were more likely to be diagnosed late than those who undertake the test by their self initiation. This finding is in line with a study in Jimma, Southwest Ethiopia, where getting tested by VCT initiation was shown to be associated with late presentation [31]. This could be due to the logic that those who undertake the test due to PITCH are most likely the ones who come for health services being infected with opportunistic infections.

Regarding to those who undertake HIV testing being initiated by families and friends; they are more likely to undertake the test earlier than those initiated by themselves since people in developing countries usually do not use to undertake the test being planned on oneself unless being initiated by somebody else and when they do it, they most likely do it while becoming older and not in early adulthood. This finding was similar with the study done in Ethiopia in south Wollo zone which has detected that those who tested with sickness/symptoms were about 2.6 times more likely to be diagnosed late [OR = 2.62, 95% CI: 1.26–5.44][28,32]. In addition, a finding from South Korea indicated that the proportion of individuals with a late diagnosis was higher in individuals tested due to clinical symptoms in public health centers compared to general health check-up. Those tested for HIV due to clinical symptoms or sicknesses or while coming for other diseases were more likely to be diagnosed late. Hence our finding was in line with this one [33] and a study in Singapore [34] where HIV testing because of illness was found to be associated with late-stage HIV disease at first diagnosis

In this study, knowledge about HIV/AIDS and testing services had been found to have significant association with late diagnosis of HIV. The multiple logistic regression analysis showed that those with high level of knowledge were 74% less likely to be diagnosed late than those with low level of knowledge. This was in agreement with a study in Venezuela [29] where low knowledge of HIV/AIDS and lack of awareness of the free HIV program was among factors associated with HIV testing. Similarly, in Uganda [35], lack of knowledge of testing services was shown to be significantly associated with delay in HIV testing. There is lack of published study done in Ethiopia assessing knowledge as a factor for late diagnosis and hence this limits us to compare our current finding in Ethiopian situation.

This case control study also has found that respondents’ internalized or perceived stigma towards HIV/AIDS was highly significantly associated with late diagnosis of HIV. Respondents with a medium and high level of internalized stigma were more likely to be diagnosed late than those with low internalized stigma [AOR = 4.94 (3.13, 7.80)] and [AOR = 16.64 (8.29, 33.40)] respectively. This finding was in line with a finding from Southern Wollo, Ethiopia [28]. This could be because that people with higher internalized stigma use to stay un-tested so that they will not be facing the stigma they perceive. In this study it was found that those who perceived HIV as stigmatizing disease were 3.1 times more likely to be late presenters than those who did not perceive HIV as stigmatizing disease[OR = 3.1, 95% CI: 1.09–8.76]. This was also consistent with a finding from Venezuela where fear for HIV-related stigma and fear for lack of confidentiality at testing site were among significant factors associated with delayed diagnosis of HIV [29]. However, in contrast to this study, stigma regarding HIV-infected people and stigma regarding HIV testing were shown to emerge as being associated with early testing in Tijuana, Mexico[36] calling for further insight in to the issue of HIV stigma. In this study, age was not found significantly associated with delay in HIV diagnosis. This contradicts with a study report by Mugavero, et. al [37, 38], where older patients were shown more likely, to be diagnosed late where as others reported that younger age to be at risk of late diagnosis [39] but the role of gender, was not investigated in our case, though the likelihood of late diagnosis have been reported commonly in men compared to women in some studies [37, 40].

This study has got some strengths and limitations of its own. To the knowledge of authors, it is the first of its kind in Northern Ethiopia, providing a basic picture of correlates of late HIV diagnosis hence, will provide a foundation for an in depth studies that would be further carried out. Moreover, its cost effectiveness and convenience for our objective which was investigating predictor variables of late diagnosis of HIV. It is also thought that the case control study design is more powerful for studying factors/predictors.

However the limitations of this study could be, related to recall, as the responses rely on recalling retrospectively, the historical events related to HIV testing in the past two years period. The social desirability and interviewers’ bias are also expected. Asking the respondents as many questions as possible, excluding some respondents with too much recall problems as well as shortening data collection period were considered to minimize recall bias. In addition, training of data collectors as well as utilization of a standard data collection tools were employed to rectify biases related to interviewers.

## Conclusions

In conclusion, knowledge and internalized stigma about HIV/AIDS are highly significantly associated with late diagnosis of HIV/AIDS. Those with lower knowledge score towards HIV/AIDS and having reported a higher internalized stigma score towards HIV/AIDS are more likely to be diagnosed late. Moreover, respondents who had undertaken HIV testing due to health care providers’ initiation were more likely to be diagnosed late than those who undertake HIV testing by their self-initiation. But subjects who had undertaken HIV diagnosis being initiated by friend, family, and socials are less likely to be late presenters. In view of this study, therefore, an informed early HIV testing practice is a matter, being affected by low awareness, as to whom should initiate testing, stigma as well as social disclosures. So promotion of general public education, enhancing education about HIV/AIDS both to increases awareness and minimize internalized stigma, encouraging people to influence their socials to undertake HIV testing are highly recommended. Self initiation for HIV testing should be encouraged to promote early diagnosis as it was found protective factor for being late. Lastly, as the above correlates may not apply for all HIV patients, we recommend future research to focus on a more detailed investigation of predictors of late HIV diagnosis in full-fledged cohorts of HIV patients as well in general population.

## References

[1] Federal Ministry of Health (FMOH). Guideline for Implementation of Antiretroviral Therapy in Ethiopia. Addis Ababa, Ethiopia, January 2005.

[2] USAID/ETHIOPIA. HIV/AIDS health profile. Addis Ababa, Ethiopia. September 2010.

[3] Kirschner D, Webb GF, Cloyd M. Model of HIV-1 disease progression based on virus-induced lymph node homing and homing-induced apoptosis of CD4 lymphocytes. J Acquir Immune Defic Syndr 2000, 24(4):352–362. 11015152 10.1097/00126334-200008010-00010

[4] Lang W, Perkins H, Anderson RE, Royce R, Jewell N, Winkelstein W. Patterns of T lymphocyte changes with human immunodeficiency virus infection: from seroconversion to the development of AIDS. J Acquir Immune Defic Syndr 1989, 2(1):63–69. 2783971

[5] Samet JH, Freedberg KA, Savetsky JB, Sullivan LM, Stein MD. Understanding delay to medical care for HIV infection: the long-term non-presenter. AIDS 2001, 15(1):77–85. 11192871 10.1097/00002030-200101050-00012

[6] Morgan D, Mahe C, Mayanja B, Okongo JM, Lubega R, Whitworth JA. HIV- 1 infection in rural Africa: is there a difference in median time to AIDS and survival compared with that in industrialized countries? AIDS 2002, 16(4):597–603. 11873003 10.1097/00002030-200203080-00011

[7] Hammer SM, Saag MS, Schechter M, Montaner JS, Schooley RT, Jacobsen DM, et., al International AIDS Society-USA panel: Treatment for adult HIV infection 2006 JAMA 2006, 296:827–843. 16905788 10.1001/jama.296.7.827

[8] Abaasa AM, Todd J, Ekoru K, Kalyango JN, Levin J, Odeke E et.,al Good adherence to HAART and improved survival in a community HIV/AIDS treatment and care programme: the experience of The AIDS Support Organization(TASO), Kampala, Uganda. BMC Health Services Research 2008, 8:241. 19021908 10.1186/1472-6963-8-241 PMC2606686

[9] Bareta JC, Galai N, Strathdee SA, Cohn S, Margolick JB, Sterling T et., al Pre and post HAART era survival and antiretroviral use among HIV infected injection drug users at different CD4 strata. Int Conf AIDS 2002, 14.

[10] Sabin CA, Smith CJ, Gumley H, Murphy G, Lampe FC, Phillips AN et.,al Late presenters in the era of highly active antiretroviral therapy: uptake of and responses to antiretroviral therapy. AIDS 2004, 18(16):2145–2151. 15577647 10.1097/00002030-200411050-00006

[11] Bedimo M. Survival from diagnosis of HIV to eligibility for ART among adult clients enrolled at Bahirdar felege hiwot referral hospital, Bahirdar, North west Ethiopia: A retrospective longitudinal study. Bahirdar, Ethiopia. (Un-published) September 2011.

[12] Marks G, Crepaz N, Senterfitt JW, Janssen RS. Meta-analysis of high-risk sexual behavior in persons aware and unaware they are infected with HIV in the United States. Implications for HIV prevention programs. J Acquir Immune Defic Syndr 2005, 39(4):446–453. 16010168 10.1097/01.qai.0000151079.33935.79

[13] Sobrino-Vegas P, García-San Miguel L, Caro-Murillo AM, Miró JM, Viciana P, Tural C, et al Delayed Diagnosis of HIV Infection in a Multicenter Cohort: Prevalence, Risk Factors, Response to HAART and Impact on Mortality. Current HIV Research, 2009, 7, 224–230. 19275591 10.2174/157016209787581535

[14] Carey MP, Schroder KEE. Development and Psychometric Evaluation of the Brief HIV Knowledge Questionnaire. AIDS Educ Prev. 2002 4; 14(2): 172–182. 12000234 10.1521/aeap.14.2.172.23902 PMC2423729

[15] Sayles JN, Hays RD, Sarkisian CA, Mahajan AP, Spritzer KL, Cunningham WE. Development and Psychometric Assessment of a Multidimensional Measure of Internalized HIV Stigma in a sample of HIV-positive Adults. AIDS Behav. 2008 9; 12(5): 748–758. 10.1007/s10461-008-9375-3 18389363 PMC2858334

[16] Castilla J, Lorenzo JM, Izquierdo A, Eugenia Lezaun M, López I, Moreno-Iribas C, et al Characteristics and trends of newly diagnosed HIV-infections, 2000–2004. Gac Sanit 2006; 20(6): 442–8. 17198621 10.1157/13096525

[17] Castilla J, Sobrino P, de la Fuente L, Noguer I, Guerra L, Parras F. Late diagnosis of HIV infection in the era of highly active antiretroviral therapy: consequences for AIDS incidence. AIDS 2002; 16(14): 1945–51. 12351955 10.1097/00002030-200209270-00012

[18] Teira Cobo R, Suárez Lozano I, Santamaría Jáuregui JM, Terrón Pernía A, Domingo Pedrol P, González García J, et al Delayed diagnosis of HIV infection in the Spanish VACH cohort [1997–2002]. Gac Sanit 2007; 21(1): 66–9. 17306189 10.1157/13099123

[19] Longo B, Pezzotti P, Boros S, Urciuoli R, Rezza G. Increasing proportion of late testers among AIDS cases in Italy, 1996–2002. AIDS Care 2005; 17(7): 834–41. 16120500 10.1080/09540120500038397

[20] Girardi E, Aloisi MS, Arici C, Pezzotti P, Serraino D, Balzano R, et al Delayed presentation and late testing for HIV: demographic and behavioral risk factors in a multicenter study in Italy. J Acquir Immune Defic Syndr 2004; 36(4): 951–9. 15220702 10.1097/00126334-200408010-00009

[21] Delpierre C, Cuzin L, Lauwers-Cances V, Marchou B, Lang T. High-Risk groups for late diagnosis of HIV infection: a need for rethinking testing policy in the general population. AIDS Patient Care STDS 2006; 20(12): 838–47. 17192149 10.1089/apc.2006.20.838

[22] Delpierre C, Dray-Spira R, Cuzin L, Marchou B, Massip P, Lang T, et al Correlates of late HIV diagnosis: implications for testing policy. Int J STD AIDS 2007; 18(5): 312–7 17524190 10.1258/095646207780749709 PMC2486458

[23] HIV Surveillance in Spain. Assessing New HIV Cases in Spain from Autonomous Communities Notification Systems: 2003–2006 periods. Available at: http://www.isciii.es/htdocs/centros/epidemiologia/epi_sida.jsp. Accessed: 03/01/11.

[24] Chadborn TR, Delpech VC, Sabin CA, Sinka K, Evans BG. The late diagnosis and consequent short-term mortality of HIV-infected heterosexuals (England and Wales, 2000–2004). AIDS 2006; 20(18): 2371–9. 17117024 10.1097/QAD.0b013e32801138f7

[25] Santos J, Palacios R, Gutierrez M, Grana M, de la TJ, Salgado F, et al HIV infection in the era of highly active antiretroviral therapy. The Malaga Study. Int J STD AIDS 2004; 15(9): 594–6. 15339366 10.1258/0956462041724253

[26] Mayben JK, Kramer JR, Kallen MA, Franzini L, Lairson DR, Giordano TP. Predictors of delayed HIV diagnosis in a recently diagnosed cohort. AIDS Patient Care STDS 2007; 21(3): 195–204. 17428187 10.1089/apc.2006.0097

[27] Lanoy E, Mary-Krause M, Tattevin P, Perbost I, Poizot-Martin I, Dupont C, et al Frequency, determinants and consequences of delayed access to care for HIV infection in France. Antivir Ther 2007; 12(1): 89–96. 17503752 10.1177/135965350701200111

[28] Abaynew Y, Deribew A, Deribe K. Factors associated with late presentation to HIV/AIDS care in South Wollo Zone Ethiopia: a case-control study. AIDS Research and Therapy 2011, 8:8 10.1186/1742-6405-8-8 21356115 PMC3058009

[29] Bonjour MA, Montagne M, Zambrano M, Molina G, Lippuner C, Wadskier FG et.al Determinants of late disease-stage presentation at diagnosis of HIV infection in Venezuela: A case-case comparison. AIDS Research and Therapy 2008, 5:6. 18416849 10.1186/1742-6405-5-6 PMC2377254

[30] Alvarez-Uria G, Midde M, Pakam R, Kannan S, Bachu L, Naik PK,. Factors Associated with Late Presentation of HIV and Estimation of Antiretroviral Treatment Need according to CD4 Lymphocyte Count in a Resource-Limited Setting: Data from an HIV Cohort Study in India. Hindawi Publishing Corporation Interdisciplinary Perspectives on Infectious Diseases Volume 2012. 10.1155/2012/293795 PMC3348638 22611389

[31] Gesesew HA, Tesfamichael FA, Adamu BT. Factors Affecting Late Presentation for HIV/AIDS Care in Southwest Ethiopia: A Case Control Study. Public Health Research 2013, 3(4): 98–107 10.5923/j.phr.20130304.03

[32] Mohammed Y, Gebrehiwot EM and Mekonnen A. Determining factors of late HIV diagnosis in Northern Ethiopia. International Journal of Current Research in Life Sciences 2015; 4 (4): 187–192

[33] Lee J, Kim GJ, Choi B, Hong K, Heo M, Kim SS et al Increasing late diagnosis in HIV infection in South Korea: 2000–2007 BMC Public Health 2010, 10:411. 20624319 10.1186/1471-2458-10-411 PMC2912814

[34] Tey JSH, Ang LW, Tay J, Cutter JL, James L. Determinants of Late-Stage HIV Disease at Diagnosis in Singapore, 1996 to 2009. Ann Acad Med Singapore 2012;41:194–99 22760716

[35] Ddamulira J. B. M, Rutebemberwa E, Tumushabe E and Nuwaha F. Factors associated with delayed diagnosis of HIV infection in Mukono district, Uganda. East African Medical Journal 2009; 86(9):411–416 21644410 10.4314/eamj.v86i9.54162

[36] Carrizosa CM, Blumberg EJ, Hovell MF, Martinez-Donate AP, Garcia-Gonzalez G, Lozada R, et al Determinants and Prevalence of Late HIV Testing in Tijuana, Mexico. AIDS PATIENT CARE and STDs 2010; 24(5):333–340 10.1089/apc.2009.0138 20438374 PMC3663452

[37] Mugavero MJ, Castellano C, Edelman D, Hicks C. Late Diagnosis of HIV Infection: The Role of Age and Sex. The American Journal of Medicine (2007) 120, 370–373. 17398235 10.1016/j.amjmed.2006.05.050 PMC3184035

[38] Kivela PS, Krol A, Salminen MO and Ristola MA. Determinants of late HIV diagnosis among different transmission groups in Finland from 1985 to 2005. HIV Medicine (2010), 11, 360–367. 10.1111/j.1468-1293.2009.00783.x 20002776

[39] Schwarcz S, Hsu L, Dilley JW, Loeb L, Nelson K, Boyd S. Late diagnosis of HIV infection: trends, prevalence, and characteristics of persons whose HIV diagnosis occurred within 12 months of developing AIDS. J Acquir Immune Defic Syndr.2006 1; 43(4):491–4. 17031318 10.1097/01.qai.0000243114.37035.de

[40] Agaba PA, Meloni ST, Sule HM, Agbaji OO, Ekeh PN, Job GC, et al Patients who present late to HIV care and associated risk factors in Nigeria. HIV Medicine (2014); 10.1111/hiv.12125 24580742

